# Supplementary material for: Quantile-dependent expressivity of serum C-reactive protein concentrations in family sets
Source: PeerJ. 2021 Feb 16;9:e10914. doi: 10.7717/peerj.10914 (PMC7894107; doi:10.7717/peerj.10914)
Supplement: Supplemental Information 1 [file peerj-09-10914-s001.docx]

Stata code used to test whether CRP concentrations are quantile-dependent

*Simultaneous quantile regression where Y is the offspring (dependent variable) and X is the parental value (independent variable)

. sqreg Y X, quantiles(5 6 7 8 9 10 11 12 13 14 15 16 17 18 19 20 21 22 23 24 25 26 27 28 29 30 31 32 33 34 35 36 37 38 39 40 41 42 43 44 45 46 47 48 49 50 51 52 53 54 55 56 57 58 59 60 61 62 63 64 65 66 67 68 69 70 71 72 73 74 75 76 77 78 79 80 81 82 83 84 85 86 87 88 89 90 91 92 93 94 95) reps(1000)

*Linear

test -45*[q050]var2 + -44*[q060]var2 + -43*[q070]var2 + -42*[q080]var2 + -41*[q090]var2 + -40*[q100]var2 + -39*[q110]var2 + -38*[q120]var2 + -37*[q130]var2 + -36*[q140]var2 + -35*[q150]var2 + -34*[q160]var2 + -33*[q170]var2 + -32*[q180]var2 + -31*[q190]var2 + -30*[q200]var2 + -29*[q210]var2 + -28*[q220]var2 + -27*[q230]var2 + -26*[q240]var2 + -25*[q250]var2 + -24*[q260]var2 + -23*[q270]var2 + -22*[q280]var2 + -21*[q290]var2 + -20*[q300]var2 + -19*[q310]var2 + -18*[q320]var2 + -17*[q330]var2 + -16*[q340]var2 + -15*[q350]var2 + -14*[q360]var2 + -13*[q370]var2 + -12*[q380]var2 + -11*[q390]var2 + -10*[q400]var2 + -9*[q410]var2 + -8*[q420]var2 + -7*[q430]var2 + -6*[q440]var2 + -5*[q450]var2 + -4*[q460]var2 + -3*[q470]var2 + -2*[q480]var2 + -1*[q490]var2 + 0*[q500]var2 + 1*[q510]var2 + 2*[q520]var2 + 3*[q530]var2 + 4*[q540]var2 + 5*[q550]var2 + 6*[q560]var2 + 7*[q570]var2 + 8*[q580]var2+ 9*[q590]var2 + 10*[q600]var2 + 11*[q610]var2 + 12*[q620]var2 + 13*[q630]var2 + 14*[q640]var2 + 15*[q650]var2 + 16*[q660]var2 + 17*[q670]var2 + 18*[q680]var2 + 19*[q690]var2 + 20*[q700]var2 + 21*[q710]var2 + 22*[q720]var2 + 23*[q730]var2 + 24*[q740]var2 + 25*[q750]var2 + 26*[q760]var2 + 27*[q770]var2 + 28*[q780]var2 + 29*[q790]var2 + 30*[q800]var2 + 31*[q810]var2 + 32*[q820]var2 + 33*[q830]var2 + 34*[q840]var2 + 35*[q850]var2 + 36*[q860]var2 + 37*[q870]var2 + 38*[q880]var2 + 39*[q890]var2 + 40*[q900]var2 + 41*[q910]var2 + 42*[q920]var2 + 43*[q930]var2 + 44*[q940]var2 + 45*[q950]var2=0

*Quadratic

test 1335*[q050]var2 + 1246*[q060]var2 + 1159*[q070]var2 + 1074*[q080]var2 + 991*[q090]var2 + 910*[q100]var2 + 831*[q110]var2 + 754*[q120]var2 + 679*[q130]var2 + 606*[q140]var2 + 535*[q150]var2 + 466*[q160]var2 + 399*[q170]var2 + 334*[q180]var2 + 271*[q190]var2 + 210*[q200]var2 + 151*[q210]var2 + 94*[q220]var2 + 39*[q230]var2 - 14*[q240]var2 - 65*[q250]var2 - 114*[q260]var2 - 161*[q270]var2 - 206*[q280]var2 - 249*[q290]var2 - 290*[q300]var2 - 329*[q310]var2 - 366*[q320]var2 - 401*[q330]var2 - 434*[q340]var2 - 465*[q350]var2 - 494*[q360]var2 - 521*[q370]var2 - 546*[q380]var2 - 569*[q390]var2 - 590*[q400]var2 - 609*[q410]var2 - 626*[q420]var2 - 641*[q430]var2 - 654*[q440]var2 - 665*[q450]var2 - 674*[q460]var2 - 681*[q470]var2 - 686*[q480]var2 - 689*[q490]var2 - 690*[q500]var2 - 689*[q510]var2 - 686*[q520]var2 - 681*[q530]var2 - 674*[q540]var2 - 665*[q550]var2 - 654*[q560]var2 - 641*[q570]var2 - 626*[q580]var2 - 609*[q590]var2 - 590*[q600]var2 - 569*[q610]var2 - 546*[q620]var2 - 521*[q630]var2 - 494*[q640]var2 - 465*[q650]var2 - 434*[q660]var2 - 401*[q670]var2 - 366*[q680]var2 - 329*[q690]var2 - 290*[q700]var2 - 249*[q710]var2 - 206*[q720]var2 - 161*[q730]var2 - 114*[q740]var2 - 65*[q750]var2 - 14*[q760]var2 + 39*[q770]var2 + 94*[q780]var2 + 151*[q790]var2 + 210*[q800]var2 + 271*[q810]var2 + 334*[q820]var2 + 399*[q830]var2 + 466*[q840]var2 + 535*[q850]var2 + 606*[q860]var2 + 679*[q870]var2 + 754*[q880]var2 + 831*[q890]var2 + 910*[q900]var2 + 991*[q910]var2 + 1074*[q920]var2 + 1159*[q930]var2 + 1246*[q940]var2 + 1335*[q950]var2 = 0

*Cubic

test -35244*[q050]var2 - 30544.8*[q060]var2 - 26109.6*[q070]var2 - 21932.4*[q080]var2 - 18007.2*[q090]var2 - 14328*[q100]var2 - 10888.8*[q110]var2 - 7683.6*[q120]var2 - 4706.4*[q130]var2 - 1951.2*[q140]var2 + 588*[q150]var2 + 2917.2*[q160]var2 + 5042.4*[q170]var2 + 6969.6*[q180]var2 + 8704.8*[q190]var2 + 10254*[q200]var2 + 11623.2*[q210]var2 + 12818.4*[q220]var2 + 13845.6*[q230]var2 + 14710.8*[q240]var2 + 15420*[q250]var2 + 15979.2*[q260]var2 + 16394.4*[q270]var2 + 16671.6*[q280]var2 + 16816.8*[q290]var2 + 16836*[q300]var2 + 16735.2*[q310]var2 + 16520.4*[q320]var2 + 16197.6*[q330]var2 + 15772.8*[q340]var2 + 15252*[q350]var2 + 14641.2*[q360]var2 + 13946.4*[q370]var2 + 13173.6*[q380]var2 + 12328.8*[q390]var2 + 11418*[q400]var2 + 10447.2*[q410]var2 + 9422.4*[q420]var2 + 8349.6*[q430]var2 + 7234.8*[q440]var2 + 6084*[q450]var2 + 4903.2*[q460]var2 + 3698.4*[q470]var2 + 2475.6*[q480]var2 + 1240.8*[q490]var2 + 0*[q500]var2 - 1240.8*[q510]var2 - 2475.6*[q520]var2 - 3698.4*[q530]var2 - 4903.2*[q540]var2 - 6084*[q550]var2 - 7234.8*[q560]var2 - 8349.6*[q570]var2 - 9422.4*[q580]var2 - 10447.2*[q590]var2 - 11418*[q600]var2 - 12328.8*[q610]var2 - 13173.6*[q620]var2 - 13946.4*[q630]var2 - 14641.2*[q640]var2 - 15252*[q650]var2 - 15772.8*[q660]var2 - 16197.6*[q670]var2 - 16520.4*[q680]var2 - 16735.2*[q690]var2 - 16836*[q700]var2 - 16816.8*[q710]var2 - 16671.6*[q720]var2 - 16394.4*[q730]var2 - 15979.2*[q740]var2 - 15420*[q750]var2 - 14710.8*[q760]var2 - 13845.6*[q770]var2 - 12818.4*[q780]var2 - 11623.2*[q790]var2 - 10254*[q800]var2 - 8704.8*[q810]var2 - 6969.6*[q820]var2 -5042.4*[q830]var2 + -2917.2*[q840]var2 - 588*[q850]var2 + 1951.2*[q860]var2 + 4706.4*[q870]var2 + 7683.6*[q880]var2 + 10888.8*[q890]var2 + 14328*[q900]var2 + 18007.2*[q910]var2 + 21932.4*[q920]var2 + 26109.6*[q930]var2 + 30544.8*[q940]var2 + 35244*[q950]var2 = 0

*Estimate linear effect

lincom -45*[q050]var2 + -44*[q060]var2 + -43*[q070]var2 + -42*[q080]var2 + -41*[q090]var2 + -40*[q100]var2 + -39*[q110]var2 + -38*[q120]var2 + -37*[q130]var2 + -36*[q140]var2 + -35*[q150]var2 + -34*[q160]var2 + -33*[q170]var2 + -32*[q180]var2 + -31*[q190]var2 + -30*[q200]var2 + -29*[q210]var2 + -28*[q220]var2 + -27*[q230]var2 + -26*[q240]var2 + -25*[q250]var2 + -24*[q260]var2 + -23*[q270]var2 + -22*[q280]var2 + -21*[q290]var2 + -20*[q300]var2 + -19*[q310]var2 + -18*[q320]var2 + -17*[q330]var2 + -16*[q340]var2 + -15*[q350]var2 + -14*[q360]var2 + -13*[q370]var2 + -12*[q380]var2 + -11*[q390]var2 + -10*[q400]var2 + -9*[q410]var2 + -8*[q420]var2 + -7*[q430]var2 + -6*[q440]var2 + -5*[q450]var2 + -4*[q460]var2 + -3*[q470]var2 + -2*[q480]var2 + -1*[q490]var2 + 0*[q500]var2 + 1*[q510]var2 + 2*[q520]var2 + 3*[q530]var2 + 4*[q540]var2 + 5*[q550]var2 + 6*[q560]var2 + 7*[q570]var2 + 8*[q580]var2+ 9*[q590]var2 + 10*[q600]var2 + 11*[q610]var2 + 12*[q620]var2 + 13*[q630]var2 + 14*[q640]var2 + 15*[q650]var2 + 16*[q660]var2 + 17*[q670]var2 + 18*[q680]var2 + 19*[q690]var2 + 20*[q700]var2 + 21*[q710]var2 + 22*[q720]var2 + 23*[q730]var2 + 24*[q740]var2 + 25*[q750]var2 + 26*[q760]var2 + 27*[q770]var2 + 28*[q780]var2 + 29*[q790]var2 + 30*[q800]var2 + 31*[q810]var2 + 32*[q820]var2 + 33*[q830]var2 + 34*[q840]var2 + 35*[q850]var2 + 36*[q860]var2 + 37*[q870]var2 + 38*[q880]var2 + 39*[q890]var2 + 40*[q900]var2 + 41*[q910]var2 + 42*[q920]var2 + 43*[q930]var2 + 44*[q940]var2 + 45*[q950]var2

*Estimate difference between 10^th^ and 90^th^ percentiles

lincom [q900]var2 - [q100]var2
